# Mindfulness May Buffer Psychological Distress in Adolescents during the COVID-19 Pandemic: The Differential Role of Mindfulness Facets

**DOI:** 10.5334/pb.1093

**Published:** 2021-11-22

**Authors:** Merle Kock, Peter Kuppens, Katleen Van der Gucht, Filip Raes

**Affiliations:** 1 Faculty of Psychology and Educational Sciences, KU Leuven, Leuven, BE

**Keywords:** Mindfulness, COVID-19, adolescents, psychological distress, decentering

## Abstract

The COVID-19 pandemic strongly impacts adolescents’ mental health, a population particularly vulnerable to mental disorders, highlighting the need to identify protective factors against COVID-19 related psychological distress to inform policies and intervention strategies. Previous research suggests that mindfulness may be a promising factor that can lower the risk of detrimental psychological consequences related to the COVID-19 pandemic. However, it is currently unknown which aspects of mindfulness contribute most to its protective effects. Moreover, previous studies mainly focused on adult samples. The present study aimed to address this gap by investigating the impact of specific mindfulness facets on adolescents’ COVID-19 related psychological functioning. 246 Dutch-speaking adolescents were recruited via social media to complete a cross-sectional online survey between June 29 and October 11, 2020. Participants were 16–18 years of age, most of them women (71%), and the majority followed the highest level of Belgian secondary education. Logistic regression analyses were performed to test the differential effects of each mindfulness facet on psychological functioning. Our results identified decentering as the facet of mindfulness that was uniquely associated with decreased worry and stress, improved mental health and quality of life, as well as with an increase in social connectedness with others following the outbreak of the COVID-19 pandemic. Unexpectedly, decentering was negatively associated with adolescents’ helping behaviour during compared to before the pandemic. Implications for research on and application of mindfulness are discussed. Taken together, these findings suggest that the facet of decentering, among all facets of mindfulness, may represent the main protective factor against psychological distress during the COVID-19 pandemic.

The COVID-19 pandemic has become a major public health crisis and is still strongly influencing our daily lives. Importantly, the pandemic does not only affect physical health but may also strongly impact mental health as a consequence of quarantine, social isolation, financial strain, and the threat of infection (Pfefferbaum & North, 2020). Data from large surveys among the Belgian population in April-December 2020 shows that the prevalence of depression and anxiety problems ranged between 14–22% and 16–23%, respectively, compared with a prevalence of 9.5% for depression and 11% for anxiety problems in 2018 (Braekman et al., 2020a, 2020b; Charafeddine, Braekman, Demarest, Drieskens, Gisle, Hermans, & Scohy, 2020; Charafeddine, Braekman, Demarest, Drieskens, Gisle, Hermans, & Vandevijvere, 2020; Gisle, Braekman et al., 2020; Gisle, Drieskens et al., 2020). In order to effectively respond to the rise in mental health problems after the outbreak of COVID-19, it is of great importance to identify protective and risk factors that determine the varied psychological reactions to the pandemic. Such research would inform policies and intervention strategies for the current and potential future large-scale infectious disease outbreaks in our globalised world and increase our scientific understanding of psychological reactions to extreme stress more generally (Holmes et al., 2020).

Across the globe, adolescents are not a focus of COVID-19 prevention strategies. However, they may be particularly vulnerable to the psychological consequences of the pandemic. In general, mental disorders are the major contributor to disease burden in young people (Patel et al., 2007). Most mental disorders emerge during adolescence; they may substantively impact other life domains and may have long-term effects on mental and physical health in adulthood. The COVID-19 pandemic further heightened the risk for adolescents to develop a mental disorder. Adolescents experienced massive disruptions to their lives, including school closure, transition to internet-based learning and social isolation from peers, which may have multiple detrimental consequences such as chronic or acute stress, worry about family and friends or about the family’s financial situation. Moreover, multiple studies identified young age as a risk factor for mental health problems during the pandemic (Charafeddine, Braekman, Demarest, Drieskens, Gisle, Hermans, & Scohy, 2020; Pierce et al., 2020). The prevalence of mental distress tripled among Belgian adolescents in the first lockdown in March 2020 compared to 2018 (Rens et al., 2021). Specifically, several Belgian health surveys between April and June 2020 reported that prevalence of depressive and anxiety disorders was continuously high among young adults (depression: 28%, anxiety: 29% in June), while prevalence was decreasing in other age groups (Charafeddine, Braekman, Demarest, Drieskens, Gisle, Hermans, & Vandevijvere, 2020). Young adults also showed the highest prevalence of negative emotions, mainly corona-related anxiety and worry, compared to other age groups (Gisle, Braekman et al., 2020). This suggests that specifically anxiety and worry are prevalent mental health problems among young people during the pandemic. Apart from direct effects on mental health, 52% of young adults reported that their life has changed a lot or completely due to the pandemic. This is particularly important because Zhu and colleagues (2020) found that not quarantine itself but the impact of the pandemic on daily life predicts general mental health problems, depression, and anxiety in adults. It is thus conceivable that the COVID-19 pandemic may have a detrimental impact on adolescents’ quality of life and may even contribute to the development of mental disorders. Finally, around 65% of Belgian young adults were dissatisfied with their social contacts (Gisle, Braekman et al., 2020). This is problematic because loneliness and low social support have been found to predict mental distress in 16 to 25-year-old Belgians during the first wave of the COVID-19 pandemic (Rens et al., 2021), possibly leading to chronic mental health problems in the long term. Taken together, these findings highlight the importance to identify protective and risk factors for psychological distress to ultimately improve mental health of adolescents following the outbreak of the COVID-19 pandemic.

One promising protective factor that may reduce the risk for the potentially detrimental psychological consequences of the COVID-19 pandemic is mindfulness. Mindfulness is defined as the ability to pay attention to one’s experience in the present moment, on purpose and without judging it (Kabat-Zinn, 1990). In both adults and adolescents, mindfulness is considered a multi-facet construct including awareness of the present moment, an open and accepting attitude, and the ability to take a step back from one’s experience without immediately reacting to it (Johnson et al., 2017). According to meta-analyses, mindfulness-based interventions can successfully reduce mental health symptoms such as stress, anxiety, and depression in both clinical and non-clinical adolescent samples (Klingbeil et al., 2017; Reangsing et al., 2021). Similarly, dispositional mindfulness may be an important factor when it comes to effective coping with the stressful pandemic situation (Weis et al., 2021). First, adolescents with high dispositional mindfulness are better able to attend to their own experiences and meaningful activities rather than responding to the rapidly changing pandemic situation. Second, adolescents with high dispositional mindfulness are more accepting of their thoughts and emotions and can therefore better cope with challenging thoughts and feelings that may otherwise be overwhelming. Finally, adolescents with high dispositional mindfulness more easily accept the pandemic and regard it as an opportunity for reflection and growth rather than a threat to their personal plans that they cannot control. The protective effect of mindfulness in the context of the pandemic has already been demonstrated in several studies. Dispositional mindfulness was the strongest predictor of psychological distress in Italian adults (Conversano et al., 2020) and showed a strong negative association with depression, anxiety, and stress in Dutch-speaking adults (Vos et al., 2021). Similarly, dispositional mindfulness significantly predicted lower anxiety, depression, and COVID-19 related traumatic stress in Chinese students (Sun et al., 2021) and mediated the impact of the COVID-19 pandemic on anxiety and depression in emerging American adults (DiFonte et al., 2020). Additionally, American students following a mindfulness-based intervention reported stable scores of anxiety and stress while the no-intervention control group showed significant increases in distress, likely caused by the outbreak of the COVID-19 pandemic (Weis et al., 2021). Change in mindfulness mediated the relationship between treatment condition and anxiety as well as stress at post-treatment, suggesting that the cultivation of mindfulness protected participants from increases in anxiety and stress due to the pandemic. Taken together, these findings suggest that dispositional mindfulness may reduce the risk for detrimental psychological consequences of the COVID-19 pandemic. However, previous studies mainly used general measures of psychological distress and it is unclear to what extent the measured psychological distress is specifically related to the pandemic. Moreover, to the best of the authors’ knowledge, previous studies mainly involved adult samples and none have been conducted in Belgian or Dutch-speaking adolescents. Given the large differences between prevalence of psychological distress in Belgian compared to British adolescents during the COVID-19 outbreak in 2020 (Pierce et al., 2020; Rens et al., 2021), it is important to investigate the protective effects of dispositional mindfulness against COVID-19 related psychological distress in a Belgian sample to provide guidance for policies and interventions specific to the Belgian population.

Furthermore, it is currently unknown which aspects of mindfulness contribute most to its protective effects against the psychological consequences of COVID-19. As mentioned above, mindfulness is a multi-facet construct. Theoretical research based on Buddhist scholarship suggests that mindfulness consists of multiple characteristics (Brown et al., 2007). Since different Buddhist traditions highlight different aspects of mindfulness, it is difficult to precisely name the facets that characterise mindfulness. Several major Buddhist traditions highlight six core facets of mindfulness while other traditions provide a more extensive set of facets. These core facets include clear awareness of one’s inner and outer experiences, non-interference with experiences, flexibility of awareness and attention, objective and non-judgmental receptivity, orientation to the present moment, and continuity of attention and awareness. To ensure coverage of all essential facets, the present study used the Comprehensive Inventory of Mindfulness Experiences (CHIME; Bergomi et al., 2014) as a measure of mindfulness. The CHIME was constructed based on items derived from all currently available adult self-report measures of mindfulness to allow for a balanced representation of all facets (Bergomi et al., 2013). Factor analysis of the CHIME identified eight facets that are central to the construct of mindfulness. These facets also apply to adolescents and include awareness of internal and external experiences, acting with awareness, an accepting and non-judgmental orientation, decentering and non-reactivity to present experiences, openness to experience, relativity of thoughts, and insightful understanding (Johnson et al., 2017). Specifically, the facets acting with awareness, acceptance/non-judgment and decentering/non-reactivity reduced the risk of increases in depression, anxiety, and weight concerns over a 12-month period in adolescents (Johnson & Wade, 2019). These findings indicate that not all mindfulness facets have an equally strong association with mental health but that some facets confer stronger protective effects against mental health issues. However, and importantly, the study by Johnson and Wade (2019) was conducted before the outbreak of the COVID-19 pandemic. It is unclear how the findings can be translated to the stress specific to the pandemic. For that reason, it is essential to investigate which mindfulness facets decrease the risk of detrimental psychological consequences of the pandemic. The gained insights would not only improve our understanding of the specific factors influencing psychological reactions to extreme stress situations but also inform the development of effective strategies to counteract the rise in mental health problems.

The present study investigates to what extent dispositional mindfulness impacts psychological functioning during the COVID-19 pandemic among 246 Dutch-speaking adolescents (16–18 years of age). Specifically, four outcome domains in relation to the COVID-19 pandemic were surveyed: symptoms of psychological distress, quality of life, helping intention/behaviour, and social connection with others. Secondly, the study aimed to identify specific facets of mindfulness that demonstrate the strongest relationship with outcomes. We hypothesised that higher dispositional mindfulness will be related to lower levels of psychological distress and higher levels of quality of life, helping intention/behaviour, and social connection with others. We had no a priori hypotheses regarding the relationship between specific mindfulness facets and outcomes.

## Methods

### Participants

Participants were 16–18 years of age (*M* = 16.73; *SD* = 0.64) and fluent in the Dutch language. Recruitment took place via social media and advertisements on Instagram. This survey was designed to test the psychometric properties of the Dutch Comprehensive Inventory of Mindfulness Experiences – Adolescents (CHIME-A), which will be reported elsewhere, and to examine the psychological impact of the COVID-19 pandemic on adolescents. The online survey was started by 529 participants but only 246 participants (71% female) completed it. The target sample size of 200 participants was determined a priori to be able to detect a small to medium correlation (ρ = .20) assuming a power of .80 and a two-tailed α=.05. Once this targeted amount of completed questionnaires has been reached, the online survey was stopped.

### Measures

To evaluate the cross-sectional effect of mindfulness on psychological outcomes during the pandemic, we conducted a survey exploring socio-demographic information, COVID-19 related psychological functioning, and dispositional mindfulness.

#### Comprehensive Inventory of Mindfulness Experiences – Adolescents (CHIME-A; Johnson et al., 2017)

The CHIME-A assesses mindfulness skills based on eight different subscales: awareness of internal experiences, awareness of external experiences, acting with awareness, an accepting and non-judgmental attitude, non-reactive decentering, openness to experiences, awareness of thought’s relativity, and insightful understanding. Its 24 items are scored on a 6-point Likert scale with higher scores indicating greater mindfulness skills. In this study, the Dutch translation of the CHIME-A was used. The translation procedure involved translation into the Dutch language by the research team, back-translation by an independent translator, and evaluation by adolescents and their teachers in correspondence with the original authors of the CHIME-A. The original questionnaire evidenced good psychometric properties in an adolescent sample (Johnson et al., 2017). Example items and indicators of internal consistency of all subscales can be found in ***Table 1***. Given that multiple arguments have been made against the use of Cronbach’s α (McNeish, 2018), internal consistency was measured using both Cronbach’s α and coefficient omega (McDonald, 1999).

**Table 1:** Example Items and Internal Consistency of CHIME-A Subscales.


SUBSCALE	EXAMPLE ITEM	α	ω

Awareness of Internal Experiences	When my mood changes, I notice it straight away.	0.43	0.44

Awareness of External Experiences	I notice details in nature (like the colour of the sky, or the shape of trees and clouds).	0.56	0.58

Acting with Awareness	I get distracted by memories or daydreams (reverse coded).	0.6	0.62

Accepting and Non-judgmental Attitude	I notice my mistakes without giving myself a hard time.	0.76	0.76

Non-reactive Decentering	When I am tangled up in uncomfortable thoughts and feelings, I notice this quickly, and can “take a step back”.	0.61	0.64

Openness to Experience	I don’t like it when I am angry or scared and try to get rid of these emotions.	0.75	0.76

Relativity of Thoughts	I realise that my point of view is not always based on facts.	0.54	0.58

Insightful Understanding	I am able to smile to myself when I notice I have made a big deal out of a small problem.	0.77	0.77


#### COVID-19 related psychological functioning

To assess different psychological outcomes related to participant’s psychological functioning during the COVID-19 pandemic, we designed a questionnaire including 12 items. The questionnaire assesses several variables during compared to before the pandemic, namely: stress and worry (“To what extent did you experience stress/did you worry during corona times compared to before the corona crisis?”), quality of life and general mental health (“How would you describe your quality of life/general mental health before/during the corona crisis?”), helping intention and behaviour (“To what extent did you feel the need to help/did you help others during corona times compared to before the corona crisis?”), and social connection to others (“To what extent did you feel connected to others during corona times compared to before the corona crisis?”). Additionally, participants were asked to select the most positive and negative emotions that they experienced during the pandemic (“What are the most positive/negative emotions you have experienced as a result of the corona crisis?”) and to rate the perceived strength of the pandemic’s effect on the world (“How big do you think the effect of the corona crisis will be on the world?”). Items were rated on a 5-point Likert scale ranging from 1 = *excellent* to 5 = *bad* (quality of life, mental health, and effect on the world) or a 7-point Likert scale ranging from 1 = *much less than before* to 7 = *much more than before* (stress, worry, helping intention, helping behaviour, social connection). The exact scales are mentioned in ***Table 2***.

**Table 2:** Descriptive Statistics of Responders’ Socio-demographic and Psychological Characteristics (*N* = 246).


VARIABLE		

*Age, Mean (SD)*	16.7	0.6

*Gender, N (%)*		

Male	66	26.8

Female	176	71.5

Other	4	1.6

*Education, N (%)*		

General education	135	54.9

Art education	7	2.8

Vocational education	11	4.5

Technical education	42	17.1

Other	51	20.7

*CHIME-A subscales, Mean (SD)*		

Internal awareness	3.7	0.6

External awareness	3.5	0.7

Acting with awareness	2.9	0.8

Acceptance	2.6	0.8

Decentering	2.8	0.7

Openness	2.9	0.9

Relativity	3.6	0.6

Insight	3.0	0.9

*Stress, N (%)*		

Much less than before COVID-19	12	4.9

Less than before COVID-19	55	22.4

A little less than before COVID-19	44	17.9

No change	31	12.6

A little more than before COVID-19	46	18.7

More than before COVID-19	48	19.5

Much more than before COVID-19	10	4.1

*Worry, N (%)*		

Much less than before COVID-19	6	2.4

Less than before COVID-19	27	11

A little less than before COVID-19	17	6.9

No change	44	17.9

A little more than before COVID-19	69	28

More than before COVID-19	64	26

Much more than before COVID-19	19	7.7

*Quality of life before the pandemic, N (%)*		

Poor	0	0

Moderate	7	2.8

Good	58	23.6

Very good	154	62.6

Excellent	27	11

*Quality of life during the pandemic, N (%)*		

Poor	18	7.3

Moderate	48	19.5

Good	94	38.2

Very good	70	28.5

Excellent	16	6.5

*Mental health before the pandemic, N (%)*		

Poor	11	4.5

Moderate	58	23.6

Good	75	30.5

Very good	83	33.7

Excellent	19	7.7

*Mental health during the pandemic, N (%)*		

Poor	36	14.6

Moderate	62	25.2

Good	80	32.5

Very good	54	22

Excellent	14	5.7

*Helping intention, N (%)*		

Much less than before COVID-19	2	0.8

Less than before COVID-19	3	1.2

A little less than before COVID-19	7	2.8

No change	80	32.5

A little more than before COVID-19	84	34.1

More than before COVID-19	64	26

Much more than before COVID-19	6	2.4

*Helping action, N (%)*		

Much less than before COVID-19	6	2.4

Less than before COVID-19	5	2

A little less than before COVID-19	8	3.3

No change	104	42.3

A little more than before COVID-19	87	35.4

More than before COVID-19	25	10.2

Much more than before COVID-19	11	4.5

*Connectedness, N (%)*		

Much less than before COVID-19	18	7.3

Less than before COVID-19	52	21.1

A little less than before COVID-19	30	12.2

No change	55	22.4

A little more than before COVID-19	60	24.4

More than before COVID-19	27	11

Much more than before COVID-19	4	1.6

*Worldwide effect, N (%)*		

No effect	1	0.4

Mild	1	0.4

Moderate	25	10.2

Large	139	56.5

Extremely large	80	32.5

*Negative emotions, N (%)*		

Worried	84	34.1

Nervous	35	14.2

Irritated	130	52.8

Anxious	49	19.9

Stressed	85	34.6

Bored	134	54.5

Isolated	133	54.1

Frustrated	93	37.8

Lonely	134	54.5

Sad	92	37.4

Angry	48	19.5

Depressed	72	29.3

*Positive emotions, N (%)*		

Hopeful	69	28

Optimistic	59	24

Rested	153	62.2

Calm	83	33.7

Relaxed	132	53.7

Relieved	33	13.4

Happy	47	19.1

Satisfied	52	21.1

Excited	34	13.8

Connected	51	20.7


##### Scale development

The instrument was developed by the authors since we were unaware of any existing measures of COVID-19 related psychological functioning for adolescents. It is based on multiple studies looking at the effect of the COVID-19 pandemic on various psychological variables such as general mental health and social isolation (Beeckman et al., 2020; Veer et al., 2021). We selected our specific outcome variables based on two aspects. First, we considered the effects of mindfulness interventions on adolescents’ psychological outcomes. A recent meta-analysis has shown that mindfulness-based interventions can reduce internalising symptoms and subjective distress, and improve pro-social behaviour in healthy and clinical adolescent samples (Klingbeil et al., 2017). Given that mindfulness interventions aim to train mindfulness skills, it is conceivable that dispositional mindfulness may influence the same psychological variables. Hence, internalising symptoms, subjective distress, and pro-social behaviour were added as outcome variables. Since the findings on the effect of mindfulness on pro-social behaviour are mixed (Poulin et al., 2021) and the pandemic caused a rise of community support initiatives, we were specifically interested in the relation between dispositional mindfulness and pro-social intentions and behaviour during the COVID-19 pandemic.

Second, we considered the psychological effects of the COVID-19 pandemic on adolescents, which may or may not be mitigated by dispositional mindfulness. As discussed above, adolescents are particularly vulnerable to the pandemic’s psychological consequences. Previous studies found that specifically anxiety and worry are prevalent among young people during the pandemic (Charafeddine, Braekman, Demarest, Drieskens, Gisle, Hermans, & Vandevijvere, 2020; Gisle, Braekman et al., 2020). Another important aspect of mental health during the pandemic is subjective stress, given that the pandemic may act as a high stress event with potential long-term mental health consequences. We therefore included a measure of worry, stress, and general mental health to assess both specific symptoms relevant in the pandemic situation and general mental distress. In addition, the pandemic strongly disrupted adolescents’ daily life and deprived them of their social contacts, with potentially detrimental consequences for quality of life and mental health (Charafeddine, Braekman, Demarest, Drieskens, Gisle, Hermans, & Vandevijvere, 2020; Gisle, Braekman et al., 2020). To assess these effects, a measure of quality of life and social connectedness was included. Taken together, we included general mental health, worry, stress, quality of life, social connectedness, and pro-social intentions and behaviour in our instrument. To assess the broader range of emotions during the pandemic that may positively or negatively impact adolescents, we measured the most prevalent positive and negative emotions that arose due to the pandemic. Finally, to estimate whether adolescents perceive the pandemic more in terms of a disruption of their individual life or as a global crisis, we assessed how strong they perceive the pandemic’s effects on the world.

To include this broad array of outcome variables, plus all questionnaires necessary for the validation of the CHIME-A, without overburdening participants, we chose to present one single item per outcome variable (two items in case of pre/post measures). Items were selected from the item pool of ongoing studies on the COVID-19 pandemic mentioned above (Beeckman et al., 2020; Veer et al., 2021) based on suitability for our sample. Moreover, single-item measures offer several advantages compared to multi-item measures. First, each additional item in a multi-item measure can increase error term correlation, meaning that each item adds only a small amount of incremental information (Drolet & Morrison, 2001). Moreover, participants tend to distinguish less between individual items when exposed to larger item sets, with earlier items more strongly influencing responses to later items. Participants are also prone to consistency motif bias, meaning they try to be consistent in their responses to similar items (Podsakoff et al., 2003). Thus, multi-item scales may lead to mindless response behaviour and response biases that do not occur for single-item measures. Single-item measures also allow participants to consider all aspects of the measured construct that are relevant to them instead of imposing an external weighting of aspects (Fuchs & Diamantopoulos, 2009). This may be advantageous when no suitable scales exist, as is the case here, and the aim is to get a broad overview over psychological effects. Unfortunately, validity and reliability of our instrument is currently unknown because these properties can only be assessed with reference to a multi-item scale that assesses the same construct (Fuchs & Diamantopoulos, 2009). However, such a scale was not available at the start of this study. Nevertheless, previous research demonstrates that single-item scales measuring stress, general mental health, and quality of life can have high reliability and validity (Ahmad et al., 2014; Boer et al., 2004; Littman et al., 2006). In conclusion, single-item scales can be reliable and valid measures of psychological functioning and offer several advantages over multi-item scales, lending support for the reliability and validity of our own instrument.

### Procedure

An online questionnaire was launched on Qualtrics (Qualtrics, Provo, UT) between June 29 and October 11, 2020, and thus after the first and before the second lockdown in Belgium. All participants provided informed consent at the beginning of the study. The study was approved by the Social and Societal Ethics Committee of the University of Leuven (G-2020-2070-R2).

### Statistical Analyses

Categorical ordinal variables were described using absolute and relative frequency distributions while continuous variables were described using means and standard deviations. Associations between all variables were assessed using Pearson’s correlation coefficient. Predictor variables that were significantly associated with a respective outcome variable at the 5% significance level based on zero-order correlations (without correction for multiple testing) were included in the main analyses. Proportional odds models were applied to examine the relationship between CHIME-A subscales and COVID-19 related psychological outcomes. This method was chosen because it is suitable for analysing ordinal outcome variables such as COVID-19 related psychological functioning, it allows to test multiple CHIME-A subscales simultaneously, and is the most widely used technique in the literature (Williams, 2016). For outcome variables that assessed psychological outcomes after the outbreak of the pandemic without relation to scores before the pandemic, scores of the respective outcome before the pandemic were included as covariates. Collinearity between predictor variables was assessed by a Variance Inflation Factor of 10 or more. The proportional odds assumption was checked in two ways. First, the brant test was applied (Brant, 1990). Second, we performed a likelihood ratio test of the proportional odds assumption for each predictor by comparing the likelihood for a proportional odds model that relies on the proportional odds assumption with the likelihood of a partial proportional odds model for which the proportional odds assumption was relaxed for a specific predictor.

Significance of predictors will be tested with likelihood ratio tests by comparing nested models with and without the predictor variables while controlling for the remaining predictor variables in the model. This method is recommended because it more accurately reflects the evidence based on the data (Christensen, 2018). Goodness of fit was assessed using Nagelkerke’s Pseudo-R^2^ (Nagelkerke, 1991). The ordinal package (Christensen, 2020) for R (R Core Team, 2021) was used to fit the models.

## Results

### Descriptive analysis

***Table 2*** displays descriptive statistics for socio-demographic and psychological variables. In line with previous research, females who follow general secondary education, the highest level of secondary education in Belgium, were more frequent among participants. 42.3% of participants reported higher stress than before the outbreak of COVID-19 and 61.7% of participants experienced increased worry compared to before the pandemic. Note, however, that 45.2% reported less stress than before the outbreak of COVID-19. Furthermore, 39.8% of participants reported poor or moderate mental health and 66% reported poor or moderate quality of life during the pandemic compared to only 28.1% and 7% of participants before the pandemic, respectively. Regarding specific negative emotions, more than 52% of participants reported that feeling bored, lonely, isolated, and irritated were the most negative emotions they experienced due to the pandemic. Notably, two of the most prevalent negative emotions point towards social deprivation. On the other hand, more than 53% of participants reported that feeling rested and relaxed were the most positive emotions they experienced due to the pandemic, while other, more high or medium arousal positive emotions, e.g. excited or happy, were less prevalent. These results indicate that a large part of participants experienced notable psychological distress following the outbreak of the COVID-19 pandemic. It is important to note, however, that this study was carried out between June and October 2020 and thus in a time period when the case numbers were low and no lockdown was in place in Belgium. Due to the nature of the questionnaire items, which were asking participants to compare their state during the COVID-19 pandemic overall to their state before the pandemic, outcomes may be influenced by the unique time of data collection or memory bias.

### Logistic regression analyses

Zero-order correlations based on Pearson’s correlation coefficient are reported in ***Table 3***. Only mindfulness subscales that correlated with an outcome variable were included in the proportional odds model for the prediction of that variable. Out of the eight mindfulness subscales, only five were included in one of the proportional odds models. Collinearity between mindfulness subscales was assessed prior to developing the multivariate model. Variance inflation factor values were < 1.5 and are thus well below the cut-off value of 10.

**Table 3:** Zero-order Correlations between all Predictor and Outcome Variables.


	VARIABLE	1	2	3	4	5	6	7	8	9	10	11	12	13	14

1	Internal awareness^a^	‒													

2	External awareness^a^	.09	‒												

3	Acting with awareness^a^	–.08	–.16*	‒											

4	Acceptance^a^	.09	–.003	.2**	‒										

5	Decentering^a^	.2**	.01	.27***	.47***	‒									

6	Openness^a^	.18**	.02	.11	.15*	–.02	‒								

7	Relativity^a^	.15*	.16*	.01	.12	.25***	–.05	‒							

8	Insight^a^	.08	.06	.12	.35***	.32***	–.05	.28***	‒						

9	Stress	–.07	.003	.03	–.17**	–.19**	–.04	–.06	–.11	‒					

10	Worry	.07	.03	–.03	–.05	–.17**	.04	–.05	–.13*	.54***	‒				

11	Quality of life^b^	.01	–.03	.1	.24***	.3***	.04	.21***	.24***	–.29***	–.26***	‒			

12	Mental health^b^	.06	–.02	.2**	.35***	.48***	.1	.12	.31***	–.37***	–.33***	.57***	‒		

13	Helping intention	.005	.08	.03	–.09	.01	–.07	.19**	.1	–.01	.02	.02	.01	‒	

14	Helping behaviour	–.05	.06	.01	–.12	–.17**	–.01	.08	.05	–.01	.1	–.07	–.08	.44***	‒

15	Connectedness	–.01	.12	.08	.09	.13*	–.05	.02	.12	–.08	–.03	.19**	.18**	.17**	.16*


*Note*: Pearson correlations without adjustment for multiple testing. ^a^ Subscales of Comprehensive Inventory of Mindfulness Experiences (CHIME-A). ^b^ Refer to values during the pandemic. * *p* < 0.05. ** *p* < 0.01. *** *p* < 0.001.

#### Mindfulness facets associated with stress and worry

The results of two separate proportional odds models predicting stress and worry are shown in ***Table 4***. The brant test of the proportional odds assumption was found insignificant for the whole model and all predictors. Likelihood ratio tests of the proportional odds assumption for each predictor were not significant, indicating that the proportional odds assumption is satisfied for all predictors in both models.

**Table 4:** Summary of proportional odds models predicting psychological outcomes.


VARIABLE	B	SE B	LR	p	OR	95% CI of OR	

						LOWER	UPPER

**DV: Stress**							

Acceptance	–0.25	0.16	2.33	0.13	0.78	0.57	1.07

Decentering	–0.37	0.19	3.99	0.05	0.69	0.47	0.99

Nagelkerke Pseudo-R^2^ = 0.05							

**DV: Worry**							

Decentering	–0.44	0.18	6.18	0.01	0.65	0.46	0.91

Insight	–0.19	0.13	1.98	0.16	0.83	0.64	1.08

Nagelkerke Pseudo-R^2^ = 0.05							

**DV: Quality of life**							

*Quality of life before COVID-19 [moderate as reference]*							

Good	–0.22	0.77	28.46	< .001	0.8	0.17	3.61

Very good/excellent	1.34	0.75			3.81	0.84	16.66

Acceptance	0.2	0.17	1.39	0.24	1.22	0.87	1.71

Decentering	0.46	0.21	4.79	0.03	1.58	1.05	2.39

Relativity	0.32	0.22	2.21	0.14	1.38	0.9	2.11

Insight	0.15	0.16	0.94	0.33	1.16	0.86	1.58

Nagelkerke Pseudo-R^2^ = 0.24							

**DV: Mental health**							

*Mental health before COVID-19 [poor as reference]*							

Moderate	0.4	0.67	23.45	< .001	1.49	0.41	5.8

Good	1.09	0.66			2.99	0.84	11.31

Very good/excellent	1.95	0.67			6.99	1.93	27

Acting with awareness	0.03	0.17	0.02	0.88	1.03	0.74	1.42

Acceptance	0.25	0.18	1.92	0.17	1.28	0.9	1.82

Decentering	1.01	0.23	20.81	< .001	2.76	1.78	4.32

Insight	0.24	0.16	2.28	0.13	1.27	0.93	1.72

Nagelkerke Pseudo-R^2^ = 0.34							

**DV: Helping intention**							

Relativity	0.66	0.2	11.15	< .001	1.93	1.31	2.86

Nagelkerke Pseudo-R^2^ = 0.05							

**DV: Helping behaviour**							

Decentering	–0.42	0.18	5.55	0.02	0.65	0.46	0.93

Nagelkerke Pseudo-R^2^ = 0.02							

**DV: Social connectedness**							

Decentering	0.36	0.17	4.43	0.04	1.43	1.02	2

Nagelkerke Pseudo-R^2^ = 0.02							


*Note*: DV = Dependent variable; *SE* = Standard Error; LR = Likelihood ratio, OR = Odds ratio; CI = Confidence Interval.

**Figure 1:**
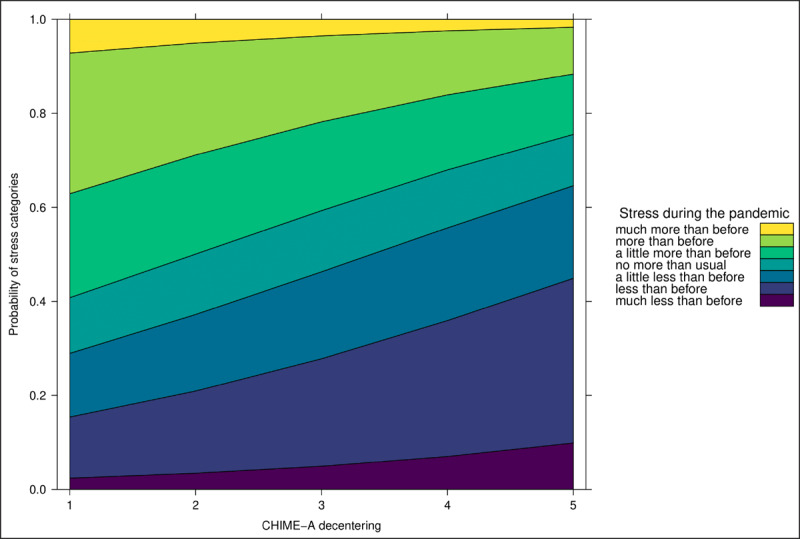
Effect of Decentering on Stress during the COVID-19 Pandemic.

**Figure 2:**
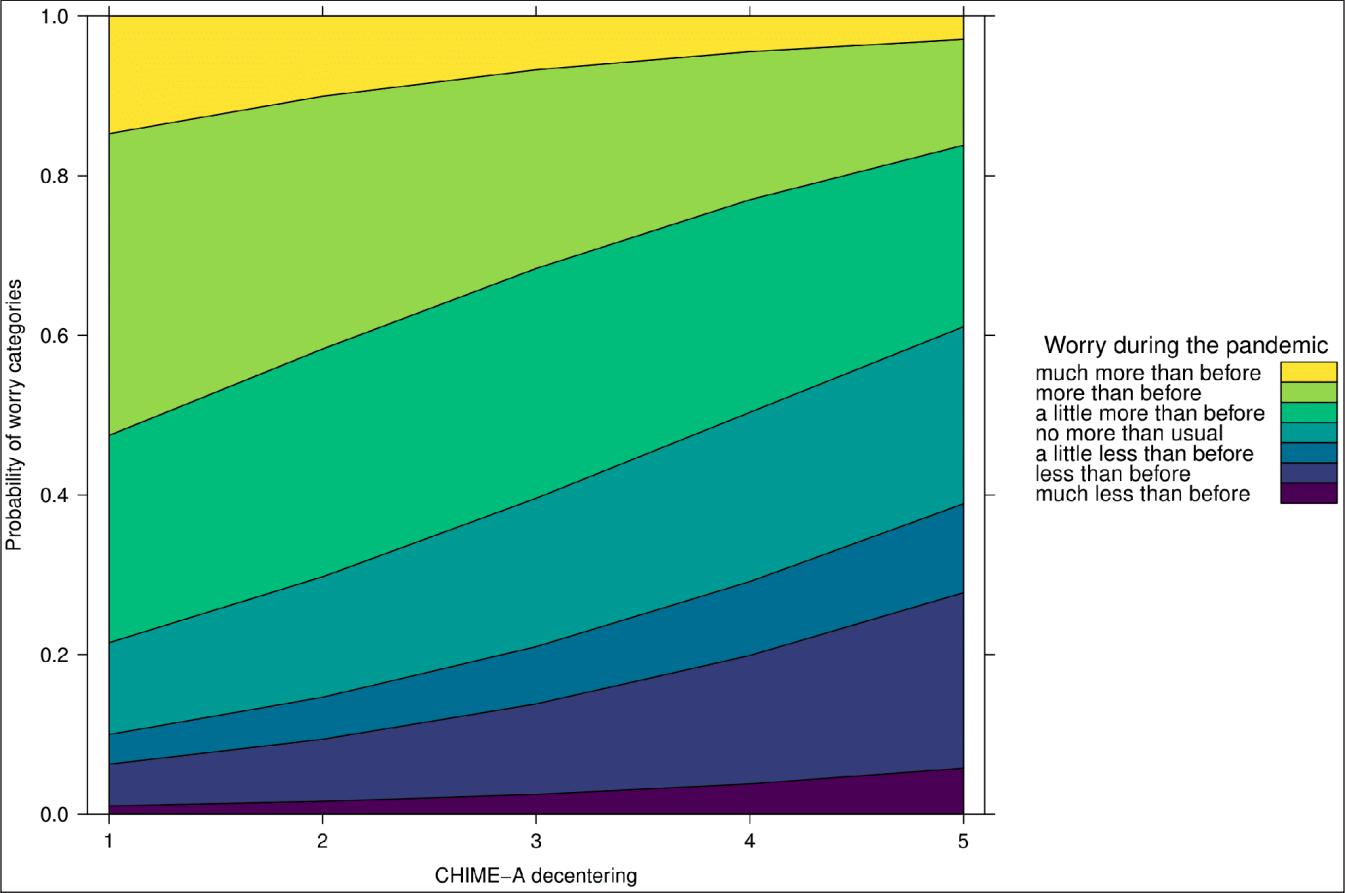
Effect of Decentering on Worry during the COVID-19 Pandemic.

As displayed in ***Figure 1***, the results show that only decentering is a significant negative predictor of stress (OR 0.69; 95% CI 0.47–0.99), meaning that with a one unit increase on the decentering subscale participants are 31% less likely to experience greater rather than unchanged or lower stress during relative to before the pandemic. As displayed in ***Figure 2***, only decentering was a significant negative predictor of worry (OR 0.65; 95% CI 0.46–0.91), meaning that with a one unit increase on the decentering subscale participants are 35% less likely to experience greater rather than unchanged or lower worry during relative to before the pandemic. Thus, the mindfulness facet of decentering had a protective effect against increases of stress and worry following the outbreak of COVID-19.

#### Mindfulness facets associated with quality of life and mental health

Results of the two separate proportional odds models predicting quality of life and mental health after the outbreak of COVID-19 are shown in ***Table 4***. Due to low frequencies of the outcome categories *very good* and *excellent* for both quality of life and mental health, these two categories were combined to increase statistical power of the overall model. The brant test of the proportional odds assumption was insignificant for the whole model and all predictors except for acceptance as a predictor of quality of life. Likelihood ratio tests of the proportional odds assumption for each predictor were insignificant, indicating that the proportional odds assumption is satisfied for all predictors in both models.

**Figure 3:**
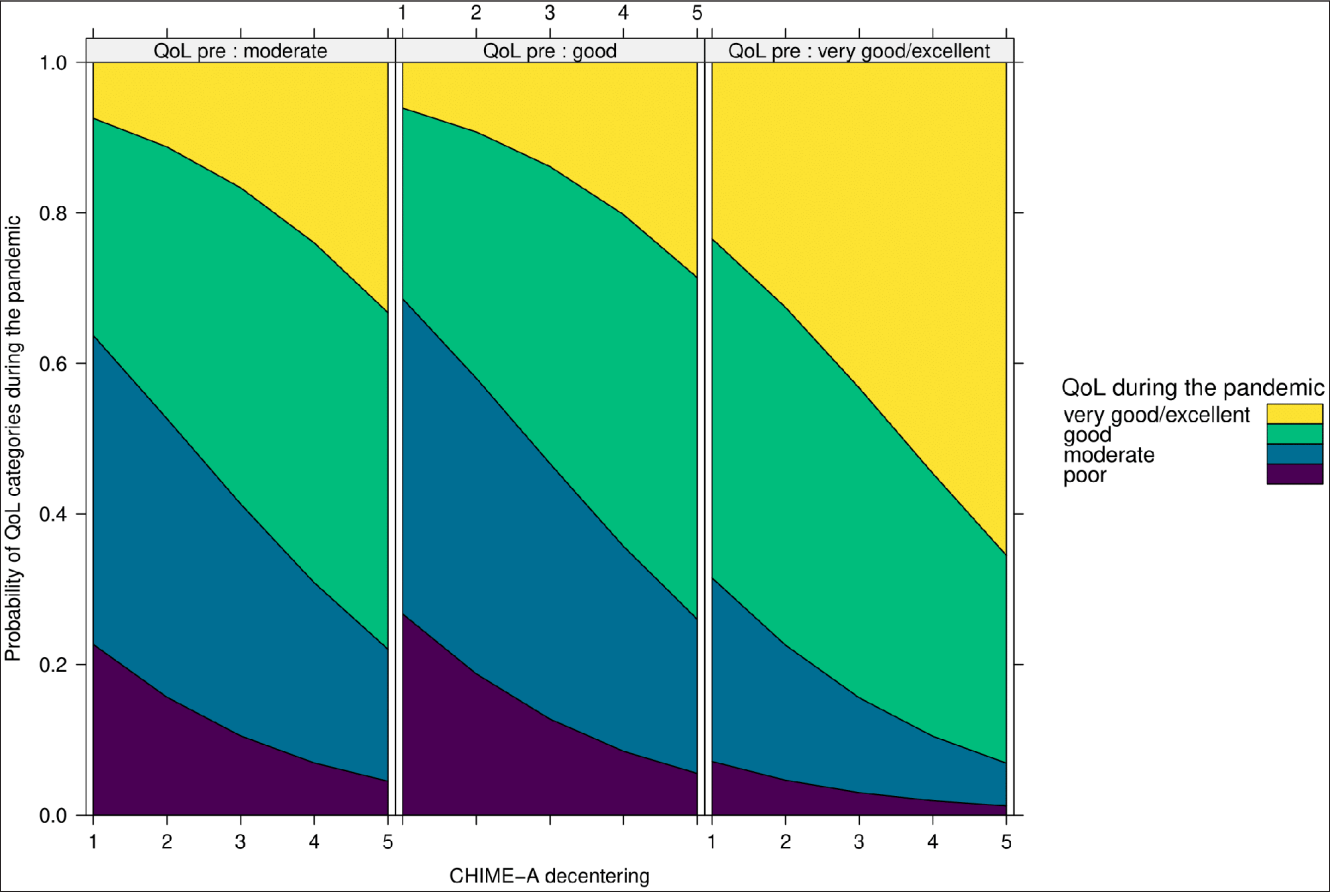
Effect of Decentering on Quality of Life (QoL) during the COVID-19 Pandemic. *Note*: QoL_pre = Quality of life before the pandemic.

**Figure 4:**
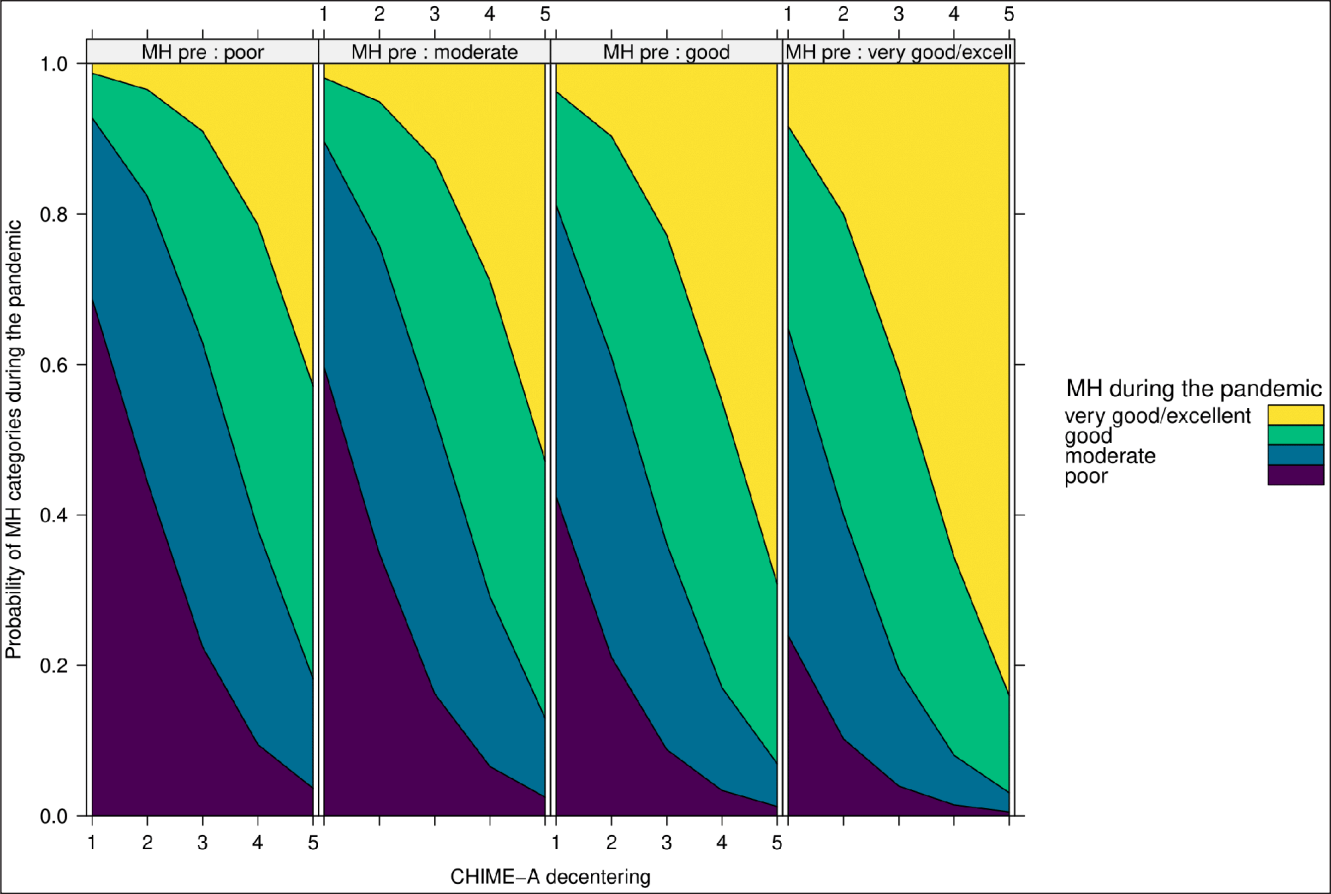
Effect of Decentering on Mental Health (MH) during the COVID-19 Pandemic. *Note*: MH_pre = Mental health before the pandemic.

As displayed in ***Figure 3***, the results demonstrate that again only decentering (OR 1.58; 95% CI 1.05 -2.39) is a significant positive predictor of quality of life during the pandemic, even when controlling for quality of life before the pandemic. With each one unit decrease on the decentering subscale, participants were 58% less likely to report at least good quality of life during the pandemic as compared to moderate or poor quality of life. As displayed in ***Figure 4***, decentering (OR 2.76; 95% CI 1.78–4.32) was found to be a significant positive predictor of mental health during the pandemic when controlling for mental health before the pandemic. Specifically, with each unit decrease on the decentering subscale participants were 2.76 times less likely to experience good or very good mental health during as compared to moderate or poor mental health. In sum, the mindfulness facet of decentering had a positive effect on adolescents’ mental health and quality of life following the outbreak of COVID-19.

#### Mindfulness facets associated with helping intention and behaviour

In ***Table 4***, results of the two proportional odds models predicting helping intention and helping behaviour are displayed. Given the low frequencies of the lower three categories indicating a decrease in helping intention/behaviour, these categories were combined in the analysis to enhance statistical power. The brant test of proportional odds assumption was insignificant for all predictors in the two models. Likelihood ratio tests of the proportional odds assumption for each predictor were not significant, indicating that the proportional odds assumption is satisfied for all predictors in both models.

**Figure 5:**
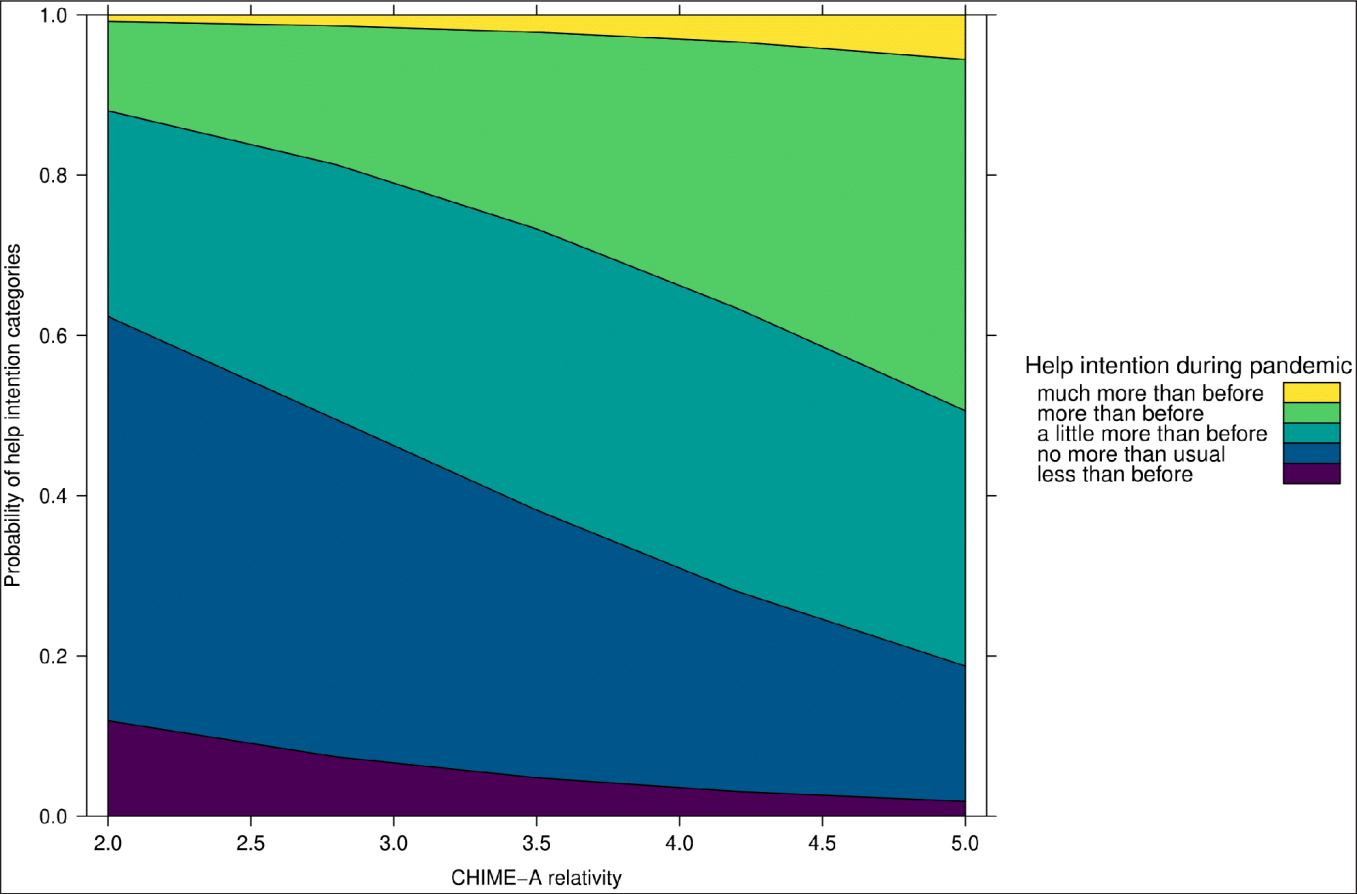
Effect of Relativity on Helping Intention during the COVID-19 Pandemic.

**Figure 6:**
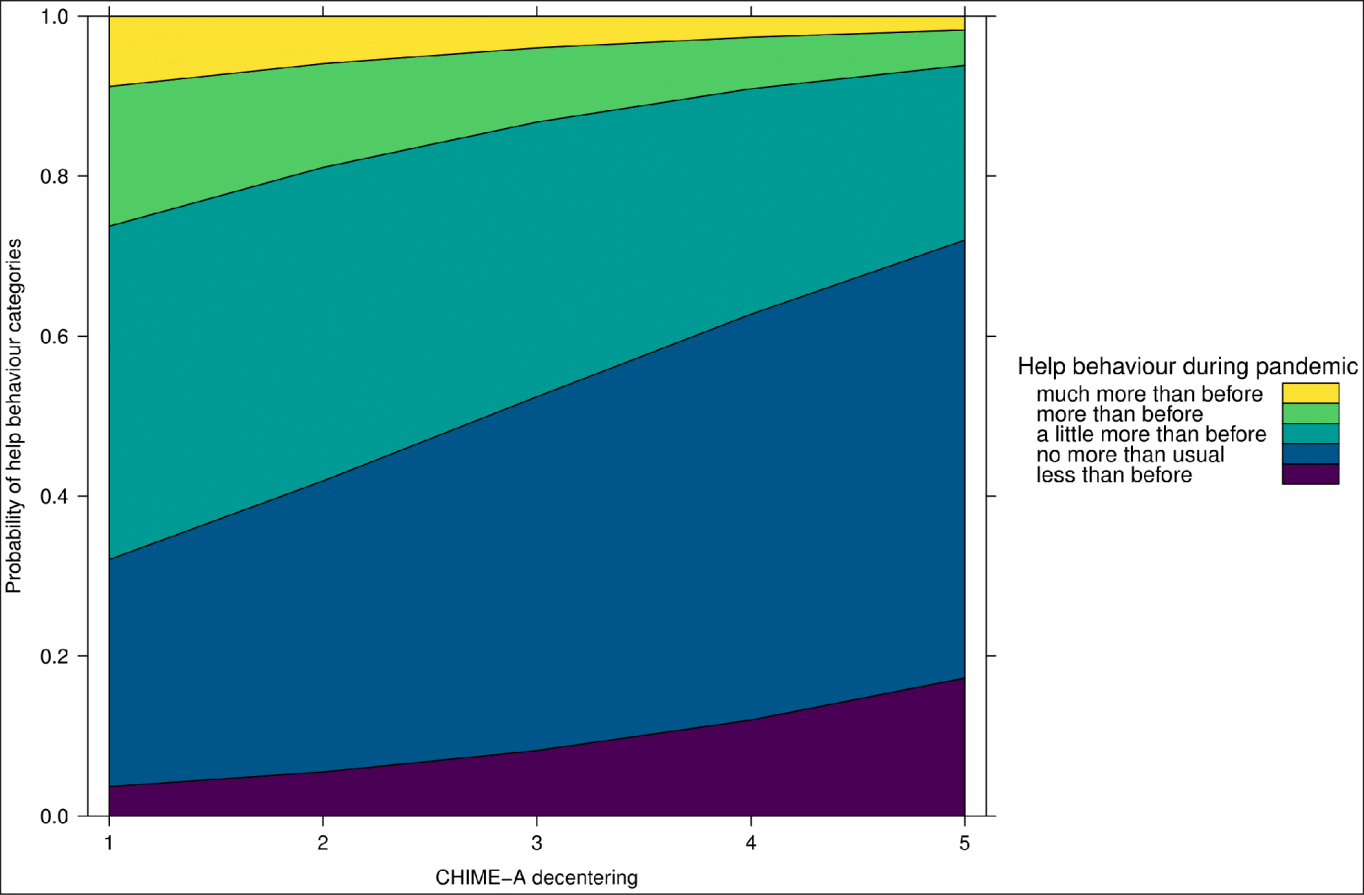
Effect of Decentering on Helping Behaviour during the COVID-19 Pandemic.

As displayed in ***Figure 5***, the results show that relativity is a significant positive predictor of helping intention (OR 1.93; 95% CI 1.31–2.86). Specifically, with every unit increase on the relativity subscale participants are 93% more likely to experience greater rather than lower or unchanged helping intention during the pandemic compared to before. As displayed in ***Figure 6***, decentering was found to significantly predict helping behaviour (OR 0.65; 95% CI 0.46–0.93), meaning that with every unit increase on the decentering subscale participants are 35% less likely to engage in more rather than unchanged or less helping behaviour during relative to before the pandemic. To conclude, the mindfulness facets of decentering had a negative effect on participants’ helping behaviour, while the facet of relativity positively influenced participants’ intention to help others during the pandemic.

#### Mindfulness facets associated with social connectedness

***Table 4*** displays the results of the model predicting social connectedness. The brant test of the proportional odds assumption was insignificant for the whole model and for the predictor. The likelihood ratio test of the proportional odds assumption for decentering was also insignificant, indicating that the proportional odds assumption is satisfied for the predictor.

**Figure 7:**
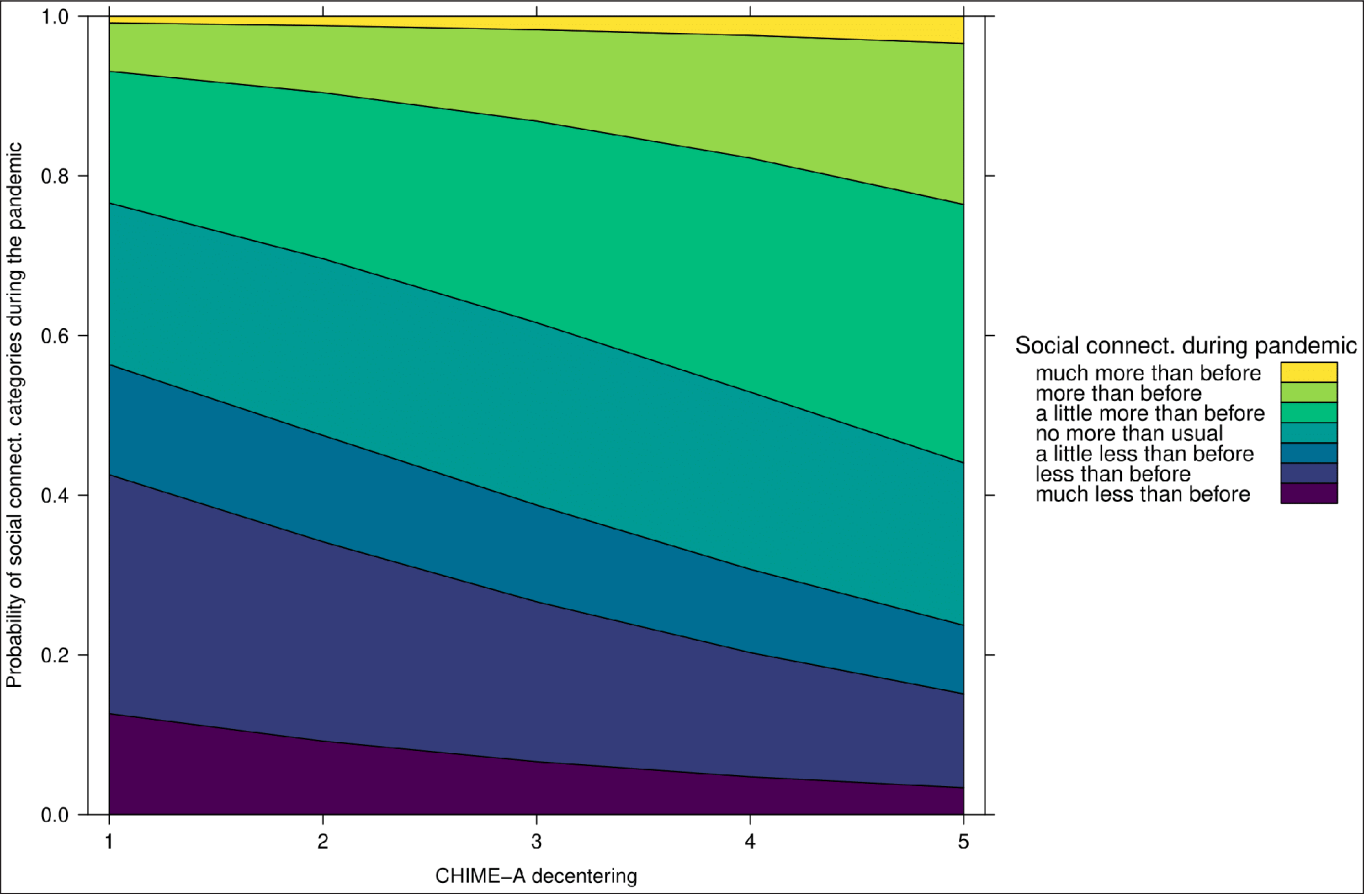
Effect of Decentering on Social Connectedness during the COVID-19 Pandemic.

As displayed in ***Figure 7***, the results show that decentering is a significant positive predictor of social connectedness (OR 1.43; 95% CI 1.02–2). Specifically, for every unit increase on the decentering subscale participants are 43% more likely to report greater social connectedness rather than unchanged or lower social connectedness during compared to before the pandemic. These results suggest that the mindfulness facet of decentering had a positive effect on adolescents’ social connectedness during the COVID-19 pandemic.

## Discussion

The present study contributes to a growing body of research looking at mindfulness as a protective factor against the adverse psychological consequences of the COVID-19 pandemic. The current work extends previous findings by focusing on the largely under-researched group of Dutch-speaking adolescents and identifying specific mindfulness facets that protect psychological functioning in the context of high stress situations such as the COVID-19 pandemic. Our results identified decentering as the facet of mindfulness that was uniquely associated with decreased worry and stress, improved mental health and quality of life, as well as with an increase in social connectedness with others following the outbreak of the pandemic. The mindfulness facet of relativity was found to be positively related to participants’ helping intention during relative to before the pandemic. Contrary to our hypotheses, decentering was negatively associated with participants’ helping behaviour such that participants with higher levels of decentering were less likely to engage in more rather than unchanged or less helping behaviour during relative to before the pandemic. Boredom, loneliness, isolation and irritation were the most prevalent negative emotions in our sample. In contrast, Gisle, Breakman and colleagues (2020) found worry and anxiety with a frequency of 6% and 27% to be the most prevalent negative emotions among Belgian adults in April 2020, while 52% of participants reported to not feel lonely. The prevalence of positive emotions was more comparable with the aforementioned study. Around 18% of adults reported feeling happy and optimistic, while in our sample 19% of adolescents reported feeling happy and 24% reported feeling optimistic. Other positive emotions were not assessed in both samples. The differences between the studies may be explained by the different time of data collection, during lockdown in April 2020 vs. between lockdowns in summer 2020, and by age differences, given that 18 to 24-year-olds had the highest prevalence of negative emotions across all age groups in the study by Gisle. Overall, our findings are in line with previous studies that identified mindfulness as protective factor against psychological distress following the outbreak of COVID-19 in adult and student populations in several countries, including Belgium and the Netherlands (Conversano et al., 2020; DiFonte et al., 2020; Götmann & Bechtoldt, 2021; Sun et al., 2021; Vos et al., 2021; Weis et al., 2021; Wielgus et al., 2020). Focusing on specific facets of mindfulness, our findings suggested that mainly the facet of decentering is responsible for the protective effects of mindfulness in adolescents. Specifically, adolescents with higher levels of decentering showed a lower risk of increased stress and worry, a higher likelihood of good or very good mental health and quality of life, and a higher likelihood of greater social connectedness to others during the pandemic relative to before. Decentering had particularly strong effects on mental health such that for every unit increase in decentering participants were three times as likely to experience good or very good mental health during the pandemic compared to moderate or poor mental health. None of the other facets of mindfulness showed a significant effect on any of these outcomes, suggesting that the facet of decentering, among all facets of mindfulness, may represent the main driver of protective effects from psychological distress during the COVID-19 pandemic.

Conceptually, decentering is comprised of three aspects: the awareness of the own subjective experience as a process of thinking, feeling and sensing (meta-awareness), the experience of internal states as separate from the self (disidentification from internal experience), and the reduced effect of thought content on other mental processes such as attention or emotion (reduced reactivity to thought content) (Bernstein et al., 2015). Meta-awareness is thought to initiate the other two components. By observing one’s experiences in the present moment and recognising that thoughts are merely interpretations that may not necessarily be true, meta-awareness allows to disidentify from one’s internal experiences and reduces automatic reactions to one’s thought content. In turn, disidentification from internal experiences and reduced reactivity to thought content influence one another and reinforce meta-awareness. By creating a distinction between the observing self and the observed experiences and reducing the habit to orient away from negative experiences, the tolerance of aversive stimuli and the ability to observe these stimuli is strengthened. In other words, disidentification from the observed experiences and reduced reactivity to negative experiences may strengthen meta-awareness of aversive stimuli. In this way, decentering enables individuals to disengage from maladaptive habitual thought patterns that promote psychological distress (Watkins, 2008). As a result, individuals with high dispositional decentering perceive the world in a different, more adaptive way that is driven by conscious choice of how to perceive and react to present experiences rather than by habitual, automatic reactions, finally promoting mental health and wellbeing (Shapiro et al., 2006).

The decentering items of the CHIME-A, the measure of mindfulness used in this study, all reflect meta-awareness and reduced reactivity to thought content and two items also reflect disidentification from internal experiences. Thus, the findings of the present study suggest that all three aspects of decentering combined are associated with mental health in adolescents, particularly in the context of high stress situations such as the COVID-19 pandemic. This is in line with and extends previous research, which found that the three aspects of decentering are associated with mental health in adults and mediated the effects of several treatments on different mental health outcomes (for a review, see Bernstein et al., 2015). With respect to internal consistency, both Cronbach’s α and ω suggest rather low levels of internal consistency for the decentering subscale of the CHIME-A (α = 0.61, ω = 0.64). This may have several reasons (Stanley & Edwards, 2016). First, it is possible that the subscale is too short to reach a higher level of reliability. However, other subscales of the CHIME-A reached acceptable levels of reliability that exceed 0.70, suggesting that the low number of items is not the sole reason for low levels of internal consistency. Instead, it is likely that the breadth of the different facets of mindfulness may have lowered the internal consistency of some CHIME-A subscales. As explained above, decentering encompasses three different aspects and can be considered a rather broad construct that may not be easily measured with high levels of internal consistency without losing its conceptual breadth. Notably, very high levels of internal consistency may not be necessary when investigating theoretical questions on a group level in a convenience sample as ours. Stanley and Edwards (2016) suggest that internal consistency levels of 0.60 or 0.70, as observed for the decentering and most other subscales in our study, are sufficient for such research purposes that do not aim to make clinical judgments like a diagnosis or treatment plan. Therefore, we conclude that most CHIME-A subscales in our sample, and specifically the decentering subscale, are sufficiently reliable to justify further inferences. However, the very low internal consistency (α and ω < .60) of the CHIME-A subscales internal awareness and external awareness may explain the lack of significant correlations between these facets of mindfulness and COVID-19 related psychological functioning.

With regard to previous research on the associations of CHIME-A subscales with mental health, our findings are partly in agreement with earlier findings from Johnson and Wade (2019) who identified the mindfulness facets of decentering but also acting with awareness and acceptance as strongest predictors of depression, anxiety, and weight concerns over a 12-month period in early adolescents. The different effects observed in our compared to the study by Johnson and Wade may be explained by the fact that decentering could be more relevant to mental health in the context of high stress situations such as the COVID-19 pandemic. This is supported by our findings from the validation of the Dutch CHIME-A (Kock et al., 2021) showing that both decentering and acting with awareness were significant predictors of depression and anxiety, and the facets of decentering, acceptance, and acting with awareness were significant predictors of stress measured by the Depression Anxiety Stress Scales (DASS-21; Lovibond & Lovibond, 1995). The DASS-21 measures depression, anxiety, and stress over the past two weeks and does therefore not specifically assess the levels of these outcomes during as compared to before the pandemic. While this study was carried out after the outbreak of the COVID-19 pandemic, data were collected mainly during the summer period when there was a limited number of COVID-19 cases and hospital admissions in Belgium and the Netherlands. Thus, DASS-21 scores were possibly less influenced by the pandemic situation than our COVID-19 related psychological functioning items that were specifically designed to assess worry and stress during the pandemic. Taken together, this suggests that the mindfulness facet of decentering specifically reduces the likelihood of psychological distress in the context of high stress situations like the COVID-19 pandemic, while the facets acceptance and acting with awareness reduce the likelihood of psychological distress more generally.

Another explanation for the different effects in our study compared to the study by Johnson and Wade (2019) may be age differences, as their study was based on early adolescents (*M_age_*=13.45 years) while our study focused on 16- to 18-year-old adolescents (*M_age_*=16.7 years). It is possible that the protective effects of specific mindfulness facets vary over the course of adolescence as some mindfulness facets have even been shown to fluctuate considerably over a 12-month period in early adolescents (Johnson & Wade, 2019). Finally, the different effects may be due to cultural differences because our sample consists of Dutch-speaking adolescents from Belgium and the Netherlands, while Johnson and Wade (2019) collected their data in Australia. Future studies may more specifically investigate the effects of different mindfulness facets on psychological distress across various age groups and cultures.

When looking at the effects of mindfulness on helping intention and behaviour, the picture is slightly more complex. Our results suggest that only the mindfulness facet of relativity enhanced the likelihood of higher helping intention during relative to before the pandemic. Relativity describes the awareness that thoughts are not facts and therefore may not necessarily be correct (Bergomi et al., 2014). In this respect, relativity is related to meta-awareness, one of the aspects of decentering, as both describe subjective experience as being based on interpretations that may not reflect reality. Due to this relativization of one’s own thoughts, adolescents with high levels of relativity are able to perceive the pandemic situation less in terms of personal loss of opportunities but more as a global problem affecting many other people who they may be able to help.

Contrary to expectations, decentering was negatively associated with helping behaviour during the pandemic. This finding is in disagreement with previous studies that found a particularly strong association between non-reactivity to thought content, one of the aspects of decentering, and social cognition skills in adults (Campos et al., 2019). One explanation might be that our measure of decentering also included disidentification from internal experience while the measure used by Campos only focused on reduced reactivity to thought content. It is possible that the disidentification from internal experience led to detachment from one’s own experience to the point of indifference in some adolescents with high dispositional decentering (Britton, 2019), which could have resulted in reduced helping behaviour during the pandemic. Given the paucity of research on the potential negative effects of mindfulness, future research should investigate for whom and to what extent mindfulness, or decentering more specifically, may have negative effects on pro-social behaviour. Such research is essential to clarify for whom and in which contexts mindfulness interventions may have unwanted effects. Another explanation for the unexpected effects of decentering on helping behaviour may be that adolescents already invested a significant amount of resources in decentering and regulating their own behaviour and emotions such that helping others may have exceeded their available resources.

The findings of the present study have to be interpreted with respect to the timing of data collection. Data were collected between June and October 2020, a period characterized by low rates of COVID-19 infections and an easing of measures related to COVID-19. Specifically, the first strict lockdown in Belgium lasted from 18th March until 4th May, while the second lockdown only applied from 2nd November 2020. Moreover, large parts of the data collection period fell within the summer holidays of Belgian schools from 1st July until 31st August. The easing of COVID-19 related measures and the occurrence of summer holidays may have influenced the ratings of adolescents in this study in a favourable direction and may not reflect psychological functioning during periods of lockdown. This is specifically relevant given the specific wording of items used to assess COVID-19 related psychological functioning. Participants were asked to rate their state during the pandemic overall until the time point of data collection compared to their state before the outbreak of the pandemic. First, these ratings may be influenced by memory bias because adolescents’ perception of their state before the pandemic and during the first lockdown may be distorted by the low COVID-19 related restrictions at time of data collection. Second, our measure of psychological functioning evaluated how adolescents perceived their situation themselves. This inherently subjective aspect of our outcome measure may have influenced our results as different adolescents may have interpreted the assessed constructs differently. Thus, our outcome measures are not directly comparable to more objective measures assessing occurrence of specific mental disorders. However, this may also be considered a strength of our outcome measure as it allows adolescents to rate a specific construct based on all aspects that are relevant to them rather than all aspects that were considered relevant by the researchers.

To provide a more objective evaluation of mental distress in our sample, our results can be compared to the general prevalence of mental disorders in Belgian adolescents and young adults. In the present study, 39.8% of adolescents reported poor or moderate mental health. This is significantly lower than the prevalence of mental distress estimated at 65.5% among 16- to 25-year-old Belgians during the first lockdown in March 2020 (Rens et al., 2021), but noticeably higher than the prevalence of mental distress of around 17.1% among 15- to 24-year-old Belgians before the pandemic in 2018 (Gisle, Drieskens et al., 2020). Hence, in line with expectations, our estimated rate of mental health problems during the pandemic falls in between the prevalence of mental distress reported during the first lockdown and the prevalence reported before the pandemic. Furthermore, 33.7% of participants in our study reported to worry more or much more during compared to before the pandemic. This finding is comparable to prevalence rates of self-reported anxiety disorders among adolescents and young adults during the pandemic, which were estimated at 25% in April, 29% in June, and 27% in September 2020 (Braekman et al., 2020b; Charafeddine, Braekman, Demarest, Drieskens, Gisle, Hermans, & Scohy, 2020; Charafeddine, Braekman, Demarest, Drieskens, Gisle, Hermans, & Vandevijvere, 2020). The prevalence of self-reported depressive disorders in the same sample was more variable at 29% in April, 28% in June, and 17% in September 2020. As a pre-COVID-19 reference, the prevalence of self-reported anxiety and depressive disorders among 15- to 24-year-old Belgians in 2018 was only 10% and 7.4%, respectively (Gisle, Drieskens et al., 2020). In conclusion, the comparison with prevalence rates of mental disorders among adolescents and young adults suggests that our estimates are lower than the prevalence of mental distress reported for March 2020 and slightly higher than the prevalence of anxiety disorders between June to September 2020. This is in line with our expectations that prevalence of mental health problems in our study, reflecting mental health during the pandemic overall until time of data collection, would be lower than prevalence rates of mental health problems during periods of lockdown but higher than prevalence rates during periods of low COVID-19 restrictions. Moreover, these findings demonstrate that prevalence rates of mental health problems assessed with our rather subjective measure of COVID-19 related psychological functioning are comparable to prevalence rates using more objective, validated assessment instruments such as the General Anxiety Disorder Questionnaire-7 (GAD-7) or the Patient Health Questionnaire-9 (PHQ-9).

### Strengths and Limitations

The present study has several strengths. Notably, this study extends previous research by investigating the differential effects of individual facets of mindfulness on psychological functioning during the COVID-19 pandemic. The outcome measures used in this study were specifically designed to assess psychological functioning in relation to the pandemic and are therefore better suitable to measure changes specific to the pandemic situation compared to generic instruments used in previous research. Finally, this study specifically focused on the protective effects of mindfulness among older adolescents, a population that is strongly impacted by the pandemic but has received little attention in previous studies on the protective effects of mindfulness (Rens et al., 2021).

Nevertheless, there are a number of limitations to be considered. First, data collection took place during the summer of 2020 when case numbers and hospitalisations related to COVID-19 were relatively low in Belgium, limiting the generalisability of our findings to other, more stressful, phases of the pandemic. Second, analyses were based on self-report data. The outcome measures specifically asked for levels on the respective outcome during compared to before the pandemic and thus may have been influenced by memory bias. Moreover, the Dutch translation of the CHIME-A used in this study had acceptable levels of internal consistency for most subscales but low internal consistency for the subscales inner awareness, outer awareness, and relativity. This may be due to the low number of only three items per subscale or due to the varying breadth of the different facets of mindfulness (Stanley & Edwards, 2016). Given that studies with the English version of the CHIME-A demonstrated higher internal consistency (Johnson et al., 2017; Johnson & Wade, 2019), it is also possible that the items and broader mindfulness facets are perceived differently by Belgian compared to Australian adolescents. Future research may clarify the cultural differences in the perception of the different facets of mindfulness. Third, our sample consisted only of 16- to 18-year-old adolescents and findings are therefore not generalisable to other age groups. Specifically, future research may focus on the differential effects of individual mindfulness facets on psychological functioning over the course of adolescence. Finally, the sample size for the present study was based on a priori power analyses for correlation analyses between the mindfulness facets and COVID-19 related psychological functioning. Thus, the sample size was not determined a priori for the logistic regression analyses performed in this study as software to perform power analyses for multinomial logistic regression is lacking.

### Implications for research and clinical interventions

The present study has both theoretical and practical implications. Our results underline the importance to distinguish between individual facets of mindfulness in future research, as they have differential effects on psychological functioning during the COVID-19 pandemic but also in non-pandemic circumstances as shown in previous studies (Johnson & Wade, 2019). Given the strong protective effects of the decentering facet of mindfulness identified in this study, it may be fruitful to investigate whether decentering can also positively influence psychological functioning in other high stress situations. Considering the unexpected negative effect of decentering on helping behaviour, however, it is necessary to clarify for whom and in which contexts decentering, both as a trait and when cultivated during mindfulness interventions, may have unwanted effects on pro-social behaviour. This is not only important to advance our theoretical understanding of potential moderators of mindfulness but also to prevent potential adverse effects.

This study also has important implications on a practical level given the detrimental psychological consequences of the COVID-19 pandemic on adolescents. Our results suggest that mindfulness-based practices and interventions with a focus on cultivating decentering may be particularly promising to support adolescents in dealing with the social deprivation and disruption of daily life caused by the pandemic. Such interventions may also prove beneficial to enhance resilience and prevent onset of chronic mental disorders in the aftermath of the COVID-19 pandemic or for similar high stress situations. Mindfulness-based interventions are particularly relevant for large-scale stress situations like a pandemic since they can be easily delivered online and are less cost-intensive, making them accessible to a large population.

## Conclusion

The present study was the first to assess the differential effects of individual mindfulness facets on psychological functioning of adolescents during the COVID-19 pandemic. The findings suggest that, among all mindfulness facets, decentering represents the main protective factor against the adverse psychological consequences of the pandemic. This is especially important when considering the strong psychological impact of the pandemic on older adolescents as noted in previous studies (Rens et al., 2021). Based on our findings, we suggest considering mindfulness-based practices and interventions with a prime focus on decentering to enhance resilience to high-stress situations such as the COVID-19 pandemic.

## Data accessibility statement

The pseudo-anonymized dataset will be made available on the OSF database *https://osf.io/dk45h/* upon publication of the validation analysis of the Dutch translation of the CHIME-A. The protocol of this study has been preregistered on the OSF database at the aforementioned link.
